# PW01-032 – FMF-like state: genetic factors unrelated to MEFV

**DOI:** 10.1186/1546-0096-11-S1-A85

**Published:** 2013-11-08

**Authors:** D Babikyan, I Jeru, B Copin, H Hayrapetyan, S Amselem, T Sarkisian

**Affiliations:** 1 Center of Medical Genetics and Primary Health Care, Yerevan, Armenia; 2 Hôpital Armand Trousseau, Paris, France

## Introduction

FMF is considered an autosomal recessive autoinflammatory syndrome caused by single gene (MEFV) mutations. Recently, it has been known that also heterozygous mutation carriers can suffer from a mild or incomplete form of FMF, named FMF-like disease. Among Armenians, who have relatively high carrier rate of MEFV mutations, single mutation has been detected in about 1/5 of symptomatic cases. Thus, one cannot exclude the influence of other modifier genes and/or environmental factors which can contribute to the variable penetrance and to the phenotypic variability of FMF-like disease.

## Objectives

The aim of our ongoing project is to better describe the genetic basis of the FMF-like condition unrelated to MEFV by identifying genetic variations in patients with single MEFV mutation.

## Methods

From records of more than 8,000 FMF patients we analyzed 105 affected heterozygous sporadic patients with definite diagnosis of FMF and extended Armenian family with variable clinical presentations. All cases were screened for full MEFV gene sequence variations and MEFV-linked five microsatellites. Whole-genome genotyping assay was applied for selected sporadic cases and 30 familial symptomatic and asymptomatic cases. Data are analyzed with Illumina GenomeStudio for LOH regions and with MERLIN for linkage analysis.

## Results

Genotype-phenotype analysis performed among one and two mutation carriers has showed high probabilities for the presence of major FMF symptoms in heterozygous individuals. Further analysis of selected 12 sporadic cases has revealed a LOH at a region encompassing genes involved in the NF-κB activation in two unrelated FMF-like sporadic patients.

Later, we authenticated autosomal dominant and autosomal recessive patterns of inheritance by finding a single mutated allele or homozygous/compound heterozygous genotypes confirmed by microsatellite analysis in several affected members of the selected family. Four of five sibs of healthy and not-related parents were carriers of a single M694V mutation, where two sibs were diagnosed with FMF and other two were asymptomatic, indicating 50% disease penetrance. Notably, the autosomal dominant inheritance was observed also in the offspring of two affected sibs with 25% (1/4) and 100% (3/3) penetrance. However, in both generations with autosomal dominant FMF we did not find a common MEFV haplotype.

## Conclusion

Parallel to the rising evidence of the association of a single MEFV mutation with only mild FMF symptoms, possibie explanation for definite phenotype of FMF in simple heterozygous patients does not exclude the association of other mutations or polymorphisms in relevant genes acting in synergy and affecting the course of FMF-like disease. Despite of the complexity of ongoing linkage analysis complicated with two consanguineous marriages and members with atypical or uncertain phenotype, co-existence of two patterns of the inheritance in the same family speculates on heterogeneous genetic basis of FMF in single mutation carriers. Preliminary finding of one candidate LOH region in sporadic cases and absence of common MEFV haplotype encourage further search of genetic variations in the genes of the inflammatory pathway acting in combination with MEFV and changing the severity of the kaleidoscopic clinical phenotype in simple carriers.

## Disclosure of interest

None declared

